# Analysis of High Sensitivity Photonic Crystal Fiber Sensor Based on Surface Plasmon Resonance of Refractive Indexes of Liquids

**DOI:** 10.3390/s18092922

**Published:** 2018-09-03

**Authors:** Xin Yan, Bin Li, Tonglei Cheng, Shuguang Li

**Affiliations:** College of Information Science and Engineering, State Key Laboratory of Synthetical Automation for Process Industries, Northeastern University, Shenyang 110819, China; 1700732@stu.neu.edu.cn (B.L.); chengtonglei@gmail.com (T.C.); lishuguang@ise.neu.edu.cn (S.L.)

**Keywords:** photonic crystal fiber, surface plasmon resonance, sensors, refractive index

## Abstract

A photonic crystal fiber (PCF) sensor based on gold nanowires able to detect changes in surface plasmon resonance (SPR) was proposed and numerically investigated through the finite element method. To facilitate real-time detection, the analyte in this sensor was located outside the optical fiber. The effects of diameters of both air hole and gold wires on the sensing characteristics of the sensor were discussed. The sensor was designed to detect liquids with refractive indexes ranging between 1.33 and 1.36. The numerical simulations indicated that sensor structure improved its functionality. The maximum spectral sensitivity reached 9200 nm/RIU over the entire refractive index range. The average spectral sensitivity was estimated to be 5933 nm/RIU, and corresponded to a sensor resolution of 2.81 × 10^−6^ RIU. These findings look very promising for future use in detection of liquid.

## 1. Introduction

In the past decade, the development of SPR sensors attracted increasing attention due to many potential future uses [1,2,3,4]. Various types of sensors have been designed and widely tested. SK Mishra et al. proposed a surface plasmon resonance-based fiber optic gas sensor for detecting gases such as ammonia, hydrogen sulfide, chlorine, hydrogen and nitrogen [5]. Y. Zhao et al. proposed a new chemical method based on silver mirror reaction for the preparation of fiber surface plasmon resonance sensing probes for liquid concentration measurement [6]. In particular, optical sensors based on photonic crystal fiber structure have attracted more attention in detection of liquid refractive indexes. SPR is among optical phenomena. Fundamentally, when light incident hits metal surfaces, the evanescent wave of the light matches the wave vector of plasma wave of the metal surface, causing resonance [7]. At this point, the surface plasma wave absorbs most energy transported by the incident light and reduces energy of the reflected light. This is also the basis of SPR applied to optical sensors [8]. However, wave vector of light is generally smaller than that of metal plasma. Hence, the use of special structure of photonic crystal fiber should match the two waves reaching the wave vector [9].

Optical fibers of conventional materials are made of SiO_2_. The refractive indexes of these materials are so large that they can not be used to detect liquids with lower refractive values. New fiber optic devices such as PCF sensors can do this [10]. They can reduce the refractive index of core by virtue of its unique periodic air-hole cladding structure, thus making the detection possible. At present, SPR sensors based on PCF mostly employ two kinds of structures.

The first is utilized to coat metal membrane inside optical fiber air holes, and selectively filling the liquid to measure. Although sensors designed by this method often have elevated sensitivities and good detection abilities, they are still limited by low mobility and slow detection speed [11,12,13]. The second way consists of depositing metal film outside the optical fiber. During real measurements, the entire body sensor is placed in the liquid to be detected and real-time detection is performed [14,15]. Filling the liquid or coating the metal film without the internal air holes means that the manufacturing process of the sensor can be simplified and is relatively easy to implement. However, the uneven thickness of the metal film when the two methods are actually produced is a problem that cannot be ignored.

In this work, we present a high sensitivity SPR sensor based on PCF structure. The two coated gold nanowires can not only reduce the influence of the uneven thickness of the gold film in the original structure, but also enhance the resonance effect and improve the sensitivity of the sensor [16,17]. Considering the reality, the axial alignment of the two gold nanowires and the PCF will cause errors. Therefore, we will explore the effects of alignment and misalignment on sensor performance in later chapters. The effect of imperfect axial alignment on sensor sensitivity is negligible within the tolerances. The high sensitivity characteristics of the resulting sensor allowed more accurate detection and determination of the refractive indexes of liquids, as well as identification of concentrations of known liquids.

## 2. Structure and Theoretical Analysis

**Figure 1:**
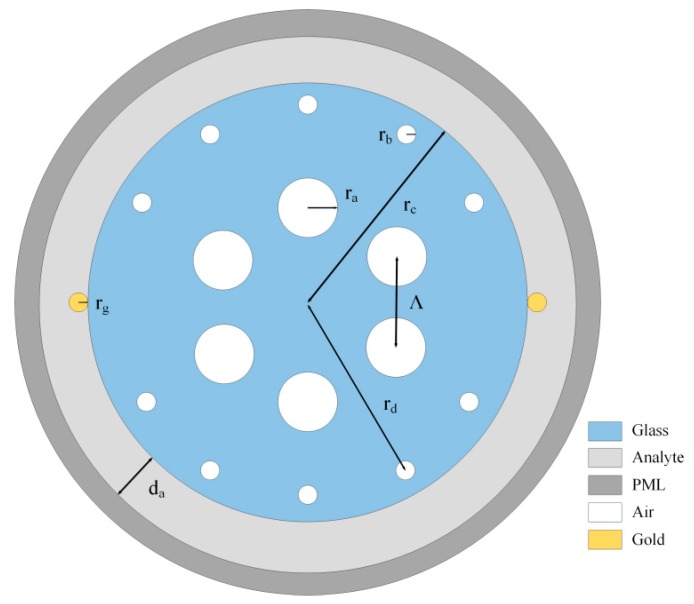
A schematic representation of the sensor structure.

A schematic representation of the proposed sensor is shown in Figure 1. The inner layer was based on silica containing six large air holes and 10 small air holes. The large air holes of the inner layer are arranged in a hexagonal shape. In addition, the small air holes in the outer layer surround the center at the same angle. A suitable optical path allows light to reach the metal surface. The entire sensor is axisymmetric. The outer layer consisted of the analyte channel coated with two gold nanowires. The distances between the inner air holes, distance from the outer air hole to the center, radius of silica and width of the analyte channel were set to Λ = 2 μm, r_d_ = 4 μm, r_c_ = 4.5 μm and d_a_ = 1 μm, respectively. 

The radii of inner air holes and outer air holes were taken as r_a_ = 0.5–0.6 μm and r_b_ = 0.2–0.4 μm, respectively. The radius of the nanowires was r_g_ = 0.2–0.32 μm. The refractive index of the analyte n varied from 1.33 to 1.36. The existence of scattering boundary conditions and perfectly matched layer (PML) can be used to absorb the energy of outward radiation [18]. The RI of background material, fused silica, is calculated by the Sellmeier Equation (1) [19]:

(1) $$n\left(\lambda \right)=\sqrt{1+\overset{m}{\underset{i=1}{\sum }}\frac{B_{i}\lambda^{2}}{\lambda^{2}-\lambda_{i}}}$$

where n is the refractive index, *λ* is wavelength in μm, *m* = 3, *B*_1_ = 0.6961663, *B*_2_ = 0.407926, *B*_3_ = 0.8974794, *λ*_1_ = 4.67914826 × 10^−3^ μm^2^, *λ*_2_ = 1.35120631 × 10^−2^ μm^2^, *λ*_3_ = 97.9340025 μm^2^. Which dielectric constant of Au is defined by the Drude-Lorentz model, Equation (2) [20]:

(2) $$\varepsilon_{m}=\varepsilon_{\infty }-\frac{\omega_{D}^{2}}{\omega \left(\omega +j\gamma_{D}\right)}-\frac{\Delta \varepsilon \omega \Omega_{L}^{2}}{\left(\omega^{2}-\Omega_{L}^{2})+j\Gamma_{L}\omega \right)}$$

where $\varepsilon_{m}$ is the permittivity of gold, $\varepsilon_{\infty }$ is the permittivity in high frequency and is approximately equal to 5.9673. $\omega_{D}$ and $\gamma_{D}$ are the plasma frequency and damping frequency, respectively. The weighting factor Δε is 1.09. ω=2πc/λ is the angular frequency, where *c* is the velocity of light, $\omega_{D}/2\pi =2113.6\;\mathrm{THz}$ and $\gamma_{D}/2\pi =15.92\;\mathrm{THz}$. The frequency and spectral width of the Lorentz oscillator are $\Omega_{L}$ and $\Gamma_{L}$, respectively, $\Omega_{L}/2\pi =650.07\;\mathrm{THz}$, and $\frac{\Gamma_{L}}{2\pi }=104.86\;\mathrm{THz}$. Confinement loss is defined by Equation (3):

(3) $$L=8.686\times \frac{2\pi }{\lambda }Im\left[n_{eff}\right]\times 10^{4}\left(\mathrm{dB}/\mathrm{cm}\right)$$

where λ represents the working wavelength and *Im*[*n_eff_*] is the imaginary part of the refractive index. The dispersion defined by Equation (4):

(4) $$D_{w}\left(\lambda \right)=-\frac{\lambda }{c}\frac{d^{2}Re_{n_{eff}}}{d\lambda^{2}}\left(\mathrm{ps}/\mathrm{km}\cdot \mathrm{nm}\right)$$

where $n_{eff}$ is the effective refractive index of the fundamental mode and $Re_{n_{eff}}$ is the real part of the effective refractive index. The hexagonal air holes should restrict most energy to the core and reduce the refractive index to it. This should meet phase matching conditions of the resonance between base model and surface plasmon. A small amount of light would pass through the air hole cladding to the surface of gold nanowires. These energies would stimulate the gold wire to produce surface plasmon resonance [7].

## 3. Results and Discussion

**Figure 2:**
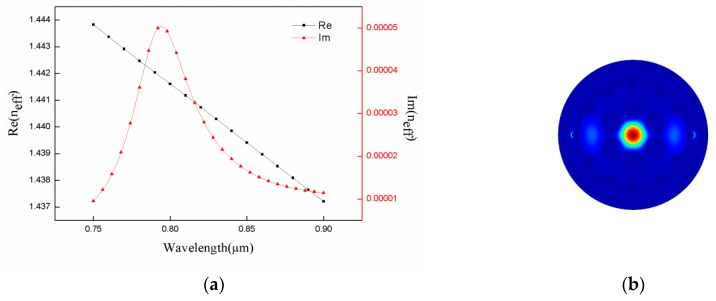
(**a**) The real and imaginary parts of the effective refractive index. (**b**) SPR appearance. Conditions: r_a_ = 0.6 μm, r_b_ = 0.2 μm, r_g_ = 0.2 μm, and n = 1.34.

**Figure 3:**
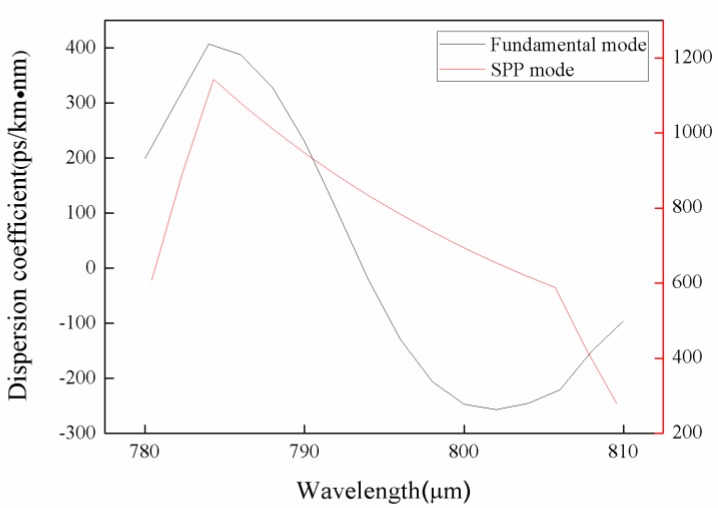
Dispersion coefficients for the fundamental mode and SPP mode. Conditions: r_a_ = 0.6 μm, r_b_ = 0.3 μm, r_g_ = 0.2 μm, and n = 1.34.

As shown in Figure 2a, the effective refractive index of fundamental mode varied with wavelength of incident light at analyte refractive index of 1.34. The black and red curves represented the real and imaginary parts of the effective index, respectively. In addition, the increase in wavelength reduced effective fraction of the refractive index (Figure 2a). The imaginary part of the effective refractive index was initially large reaching up to 794 nm then followed by a decreasing trend. The distribution of the electric field at the peak is depicted in Figure 2b. At peak value, the fundamental mode was coupled with SPP mode to yield the strongest intensity. Y. Lu et al. have proposed the polarization effects on the excitation of plasmons and their impact on the performance of the structure as a refractive index sensor. They note that the silver nanowire surface has several waveguide modes that result in several peaks. The coupling strength between the higher order mode and the core guided mode is weak. However, the coupling strength between the fundamental mode and the core guided mode is strong enough for sensing [16]. Gold and silver are similar in SPR. Figure 3 shows the dispersion coefficients for the fundamental mode and SPP mode.

**Figure 4:**
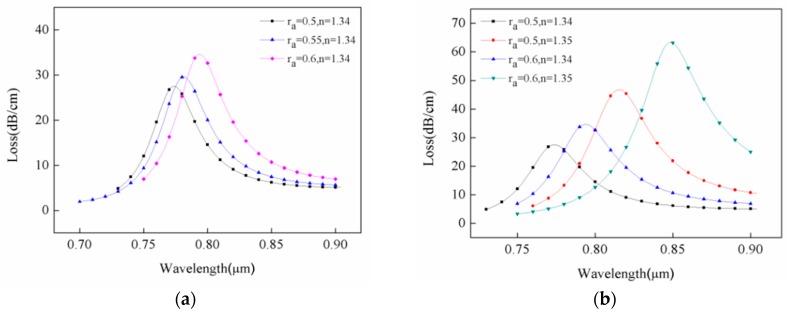
(**a**) Loss spectra of the same refractive index under different inner air hole radii. (**b**) Effects of changes in radius of inner air hole on resonance wavelength under different refractive indexes. Conditions: r_b_ = 0.2 μm and r_g_ = 0.2 μm.

The theoretical analyses and simulations revealed that changes in structural parameters of the sensor had great influences on its characteristics. The effects of varied radius of inner air hole cladding on size of the confinement loss and location of loss peak are illustrated in Figure 4a. The changing range of the air hole was estimated to 0.5–0.6 μm. At refractive index of analyte in the channel of 1.34 (Figure 4a), the confinement loss at the peak value gradually increased as radius of the inner air hole rose to yield changes of about 7 dB/cm. The latter was caused by enhanced resonance intensity. Meanwhile, the resonant wavelength shifted from 774 nm to 794 nm as radius rose from 0.5 μm to 0.6 μm, with changes of 20 nm in long wavelength direction. To gain a better understanding of the influence of radius change of the inner air hole on wavelength and loss in resonance peak under different refractive indexes, a specific comparison was performed and the data are compiled in Figure 4b. At refractive indexes of 1.34 and 1.35, the resonant wavelength of air hole radii at 0.5 μm and 0.6 μm were estimated to 42 nm and 54 nm, and peak loss changes reached around 19 dB/cm and 29 dB/cm, respectively. It can be seen that the size of the inner air hole directly affects the loss of the fiber and the spectral sensitivity of the sensor. By changing its radius, we can get better sensitivity and proper fiber loss.

**Figure 5:**
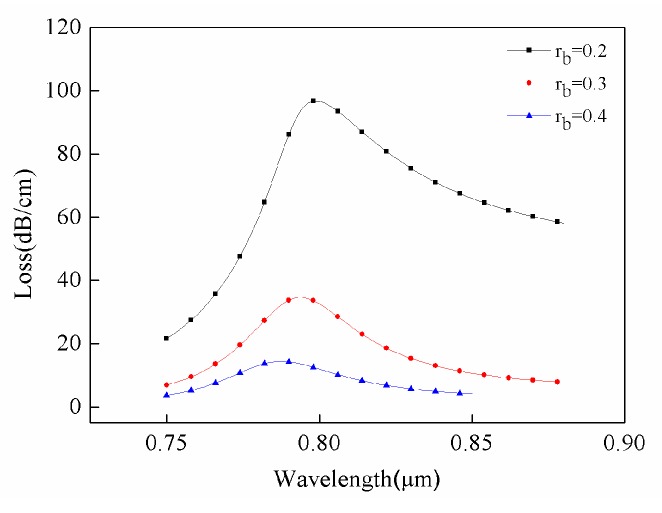
Loss spectra of different outer air hole radii. Conditions: r_a_ = 0.6 μm, r_g_ = 0.2 μm, and n = 1.34.

Figure 5 shows the effect of changes in small air-hole radius on sensor restraint loss. As radius rose, the restraint loss of the sensor significantly decreased. In particular, when the small pore radius increased from 0.2 μm to 0.4 μm, the confinement loss declined by about 82 dB/cm. The size of small pores affected the refractive index of optical fiber cladding. It should be noted that the refractive index was directly related to the binding ability of cladding to light. When the binding capacity enhanced, the loss in sensor sensitivity reduced.

**Figure 6:**
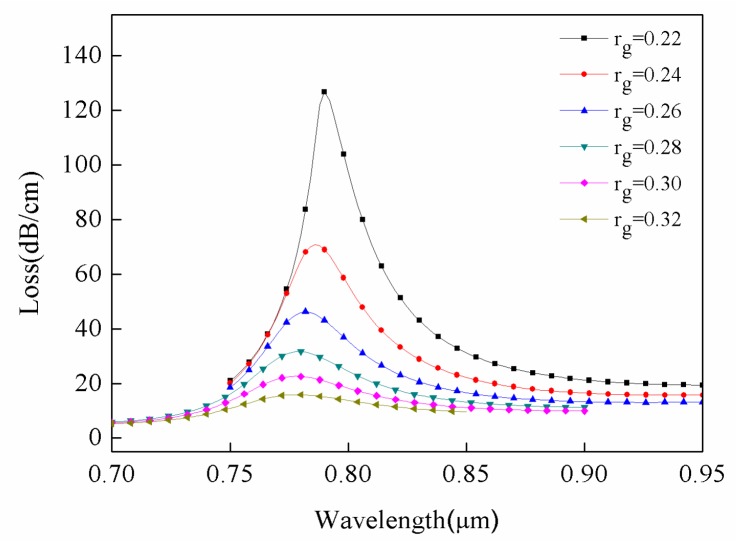
Loss spectra of the different radius of gold wire. Conditions: r_a_ = 0.6 μm, r_b_ = 0.2 μm, and n = 1.34.

The radii of gold nanowires are also related to the resonance intensity. As shown in Figure 6, when the radius of the gold wire varied between 0.22 μm and 0.32 μm, the loss in sensor intensity reduced by about 110 dB/cm. In addition, the resonance wavelength shifted slightly to shorter wavelengths and moved from 798 nm to 788 nm. 

**Figure 7:**
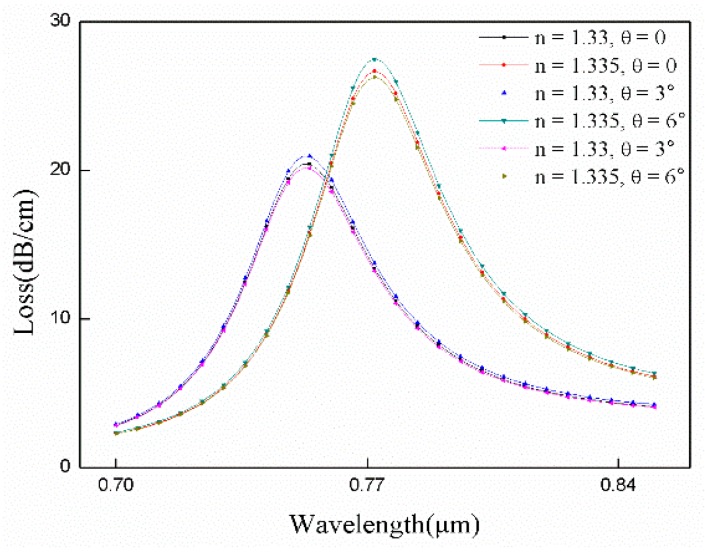
Loss spectra of the gold nanowires at different angles. Conditions: r_a_ = 0.6 μm, r_b_ = 0.2 μm, and n = 1.34.

In order to verify whether the alignment accuracy of the gold wires have an effect on the sensor performance, we rotate the gold wire on one side around the center by θ degrees. The results in Figure 7 show that the formant does not move. Therefore, the alignment accuracy affects the loss of the fiber but does not affect the sensitivity of the sensor. The number of gold nanowires also affects the loss of the fiber. Optimize their radius and number to get better sensor performance. If the number and radius are increased, the loss under the same conditions will be greater.

For sensors, sensitivity is an important performance indicator. The detection of displacement based on wave crest and detection of amplitude at specific wavelengths are methods employed to evaluate the sensitivity. Here, the different refractive indexes of the analytes can induce movement of the formant. The spectral wavelength sensitivity (*S_λ_*) based on this phenomenon can be expressed by Equation (5):

(5) $$S_{\lambda }=\frac{{\Delta \lambda }_{peak}}{\Delta n}\left(\mathrm{nm}/\mathrm{RIU}\right)$$

Δ*λ_peak_* intensity is used to define the displacement of resonance wavelength, and Δ*n* is employed for changes in the refractive index [21]. At wavelength resolution of the spectrometer set to Δ*λ_min_* = 0.1 nm, the corresponding refractive index resolution (R) of the sensor can be expressed by Equation (6):

(6) $$R=\Delta n\times \frac{{\Delta \lambda }_{min}}{{\Delta \lambda }_{peak}}$$

The amplitude sensitivity *S_a_* can be obtained using Equation (7):

(7) $$S_{a}=-\frac{1}{\alpha \left(\lambda ,n\right)}\times \frac{\partial \alpha \left(\lambda ,n\right)}{\partial n}\left({\mathrm{RIU}}^{-1}\right)$$

where *α*(*λ*,*n*) and *∂n* are the current loss of refractive index and amount of change in refractive index, respectively. *∂α*(*λ*,*n*) is the loss difference under two different refractive index conditions at the same wavelength.

**Figure 8:**
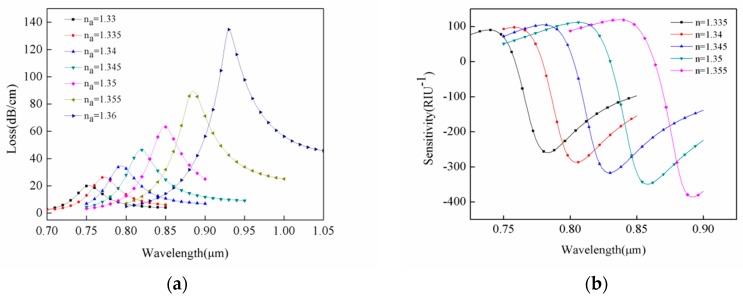
(**a**) Changes in loss peak with different analyte refractive indexes. (**b**) Changes in amplitude sensitivity at different analyte refractive indexes. Condition: r_a_ = 0.6 μm, r_b_ = 0.3 μm, and r_g_ = 0.2 μm.

Figure 8a represents the loss spectrum of liquid in the channel filled with media at different refractive indexes and Figure 8b represents amplitude sensitivity of the refractive indexes of different analytes. As shown in Figure 8a, the refractive index of the analyte varied from 1.33 to 1.36 (Δ*n* = 0.03). In addition, the peak shifted toward the long wavelength direction, and displacement amount was estimated to 178 nm (*Δλ_peak_*). The average wavelength sensitivity of the sensor obtained by Equations (2) and (3) was 5933 nm/RIU, and refractive index resolution was 2.81 × 10^−6^ RIU. When the refractive index of analyte changed from 1.355 to 1.36 (Δ*n* = 0.005), the resulting displacement amount was 46 nm, and maximum wavelength sensitivity of the sensor was 9200 nm/RIU. On the other hand, wavelength of 892 nm yielded an amplitude sensitivity of 385 RIU^−1^ at analyte refractive index of 1.355 (Figure 8b). Hence, a sensor resolution of about 2.6 × 10^−5^ RIU could be obtained by detecting 1% change in transmission intensity.

**Figure 9:**
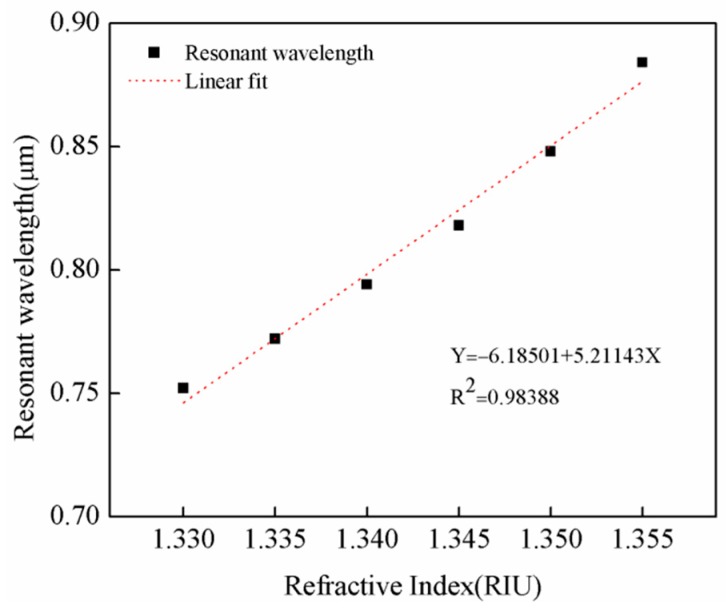
The fitting line of resonance wavelengths as a function of refractive index of the analyte.

A good linear response is important for sensor performance as it allows determination of unknown values by simple data extrapolation. Figure 9 depicts the fitted straight line of resonant wavelength of analytes with different refractive indexes ranging from 1.33 to 1.355. The regression equation of the linear fitting was determined as: Y = −6.18501 + 5.21143X, where Y and X represent the resonance wavelength and analyte refractive index, respectively. The linear regression of the fitted line was calculated as: R^2^ = 0.98388. In addition, the independent variable refractive index could fully be explained by the resonant wavelength of the dependent variable. This suggested the good linearity response of the sensor and high accuracy for determination of liquid refractive indexes.

## 4. Conclusions

A PCF sensor based on surface plasmon resonance for detection of liquids refractive indexes was successfully designed. The numerical analyses based on finite element method were employed to explore both structure and performance of the sensor. The set of conditions perfectly matched the layers and boundary scattering features to absorb emitted energy out of the system. By optimizing the structural parameters for higher sensitivities, the effects of different air hole radii, gold line radii, and analyte refractive indexes on confinement loss were all analyzed. After comprehensively analyzing the effects of air hole radius and gold wire radius on sensitivity and loss, we determined the sensor parameters in Figure 8 to achieve optimality. Simulation results show that the average values of wavelength sensitivity and sensor resolution for the analytes with refractive indexes ranging from 1.33 to 1.36 were estimated as 5933 nm/RIU and 2.81 × 10^−6^ RIU, respectively. Wavelength and amplitude sensitivities up to 9200 nm/RIU and 385 RIU^−1^ with corresponding sensor resolutions of 2.81 × 10^−6^ RIU and 2.6 × 10^−5^ RIU were obtained. Overall, the proposed sensor had simpler structure and yielded high sensitivity. Besides, it could greatly reduce errors during measurements. According to our analysis, SPR-based PCF sensors have great potential and application value in the direction of low refractive index liquid detection.
